# Integration of cortical population signals for visual perception

**DOI:** 10.1038/s41467-019-11736-2

**Published:** 2019-08-23

**Authors:** Ariana R. Andrei, Sorin Pojoga, Roger Janz, Valentin Dragoi

**Affiliations:** Department of Neurobiology and Anatomy, McGovern Medical School, University of Texas–Houston, Houston, TX 77030 USA

**Keywords:** Neuroscience, Psychology, Mathematics and computing

## Abstract

Visual stimuli evoke heterogeneous responses across nearby neural populations. These signals must be locally integrated to contribute to perception, but the principles underlying this process are unknown. Here, we exploit the systematic organization of orientation preference in macaque primary visual cortex (V1) and perform causal manipulations to examine the limits of signal integration. Optogenetic stimulation and visual stimuli are used to simultaneously drive two neural populations with overlapping receptive fields. We report that optogenetic stimulation raises firing rates uniformly across conditions, but improves the detection of visual stimuli only when activating cells that are preferentially-tuned to the visual stimulus. Further, we show that changes in correlated variability are exclusively present when the optogenetically and visually-activated populations are functionally-proximal, suggesting that correlation changes represent a hallmark of signal integration. Our results demonstrate that information from functionally-proximal neurons is pooled for perception, but functionally-distal signals remain independent.

## Abstract (web summary)

Primary visual cortical neurons exhibit diverse responses to visual stimuli yet how these signals are integrated during visual perception is not well understood. Here, the authors show that optogenetic stimulation of neurons situated near the visually‐driven population leads to improved orientation detection in monkeys through changes in correlated variability.

## Introduction

**Fig. 1:**
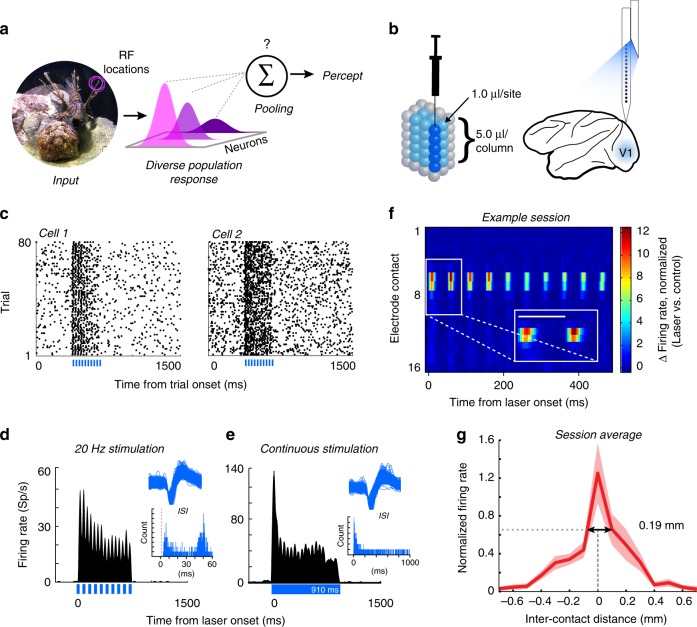
Optogenetic targeting of localized neural subpopulations. **a** Sensory stimuli activate populations of diversely tuned neurons, whose activity is integrated according to unknown pooling rules to generate sensory percepts. **b** Virus injections and recordings were aligned with a custom grid. We injected 1.0 µl of virus in V1 at five cortical depths in a columnar fashion. Electrophysiological recordings were performed using laminar electrodes tightly coupled to a fiber optic for light delivery. **c** Raster plots from two example V1 neurons showing increased activity in response to pulsed light stimulation (laser timing shown in blue at the bottom of each plot), while the monkey fixated on a central point on a monitor. **d**, **e** To confirm the absence of optical artifacts, we compared the waveforms and firing rates of a sample of neurons (one example neuron shown) during pulsed (**d**) and continuous (**e**) laser stimulation. Upper insets show the distinct action potential waveforms recorded in each respective experiment. Lower insets show the interspike intervals (ISIs) in each stimulation condition. Vertical red dashed line denotes the 1 ms refractory period. Optical artifacts, when present, occur only at the onset and offset of optical stimulation^30^ and do not exhibit typical action potential waveform shapes. During pulsed stimulation (**d**, lower inset) the responses are distributed around the duration of each laser cycle period, without an intermediate peak at 10 ms corresponding to offset (width) of each individual laser pulse. Similarly during continuous stimulation (**e**, lower inset) there is no second peak that would correspond with laser offset. **f** Distribution of optically induced activity across electrode contacts for one example session. Inter-contact spacing is 100 µm (most superficial channel is labeled “1”). Inset shows blow up of the first two laser pulses (scale bar represents 50 ms). **g** Spatial spread of laser activation. Normalized firing rates were aligned with the channel showing the largest change in laser-induced activity, interpolating for distances between channels, and averaged across sessions. Negative inter-contact distances represent channels above (closer to the surface of the brain) the reference contact. Dashed lines and arrows show the spatial spread of laser activity at full width at half maximum. Error represents s.e.m.

Our everyday visual experience relies on the ability to detect subtle changes in the structure of natural scenes. The brain must accomplish this task by combining signals originating from neural populations in sensory areas that respond to incoming stimuli, and then generate behavioral responses based on aggregate neural activity. Although primary visual cortex (V1) is necessary for normal vision^1,2^, neural responses in V1 correlate only poorly with perceptual reports^3^. This suggests that V1 activity itself must be sampled by downstream areas in order to generate visual percepts. However, even within cell populations that share overlapping receptive fields, responses of V1 neurons are highly heterogeneous, due to differences in preferred stimulus features^4–6^. Hubel and Wiesel^4^ famously hypothesized that a functional unit of cortex, containing all the necessary machinery to analyze a portion of visual space was contained within a “hypercolumn” spanning 1 mm^2^, but little evidence has emerged to support whether perceptual processes are able to integrate the information spread across such a hypercolumn. The rules governing how downstream areas parse and integrate such diverse signals from early sensory cortex to guide perceptual decisions are unknown (Fig. 1a).

Understanding the basic principles underlying how perceptually relevant signals are combined lies at the very core of any theory linking neuronal responses to behavior. Although pooling rules across cortical populations have been the subject of theoretical debate over the past few decades, they have been difficult to test experimentally. Indeed, it has been proposed that downstream areas exclusively interrogate the responses of neurons best tuned to the features of incoming stimuli^7–12^. However, sensory cortical neurons typically have bell-shaped tuning curves, and are often activated by stimuli that differ widely from their preferred feature, hence identifying the neurons that participate in coding is not trivial. An alternative strategy has been to query the responses of a broader population of cells, including neurons that do not prefer the stimulus, and pool them using a set of weights^13–18^. These approaches, however, can only narrow down the spectrum of possible pooling rules, but cannot definitively evaluate whether the cortex actually implements one rule versus another. For example, employing optimal pooling strategies to identify stimuli has often led to results outperforming the animal’s behavior^8,10,15,19^. This suggests that the actual mechanism of signal integration from early sensory areas relies on a different principle.

Experimental studies have directly addressed the issue of signal integration relevant for perception by employing causal manipulations using lesion, pharmacological, and electrical stimulation techniques^20–24^. However, lesion and pharmacological studies have reported effects that are often irreversible, and the associated techniques have limited spatial and temporal precision. While these techniques were successful in identifying the brain areas contributing to visual perception, they could not be adequately used to examine how heterogeneous signals originating from nearby neural populations are combined to influence behavior. In contrast, electrical stimulation techniques have better temporal precision, and were previously used to demonstrate a bias in behavioral choices when small currents were injected into sensory cortical areas^25,26^. However, besides the low spatial precision of electrical stimulation and lack of cell-type specificity, a key limitation of this technique is the inability to simultaneously record the relevant neural signals emitted when electrical pulses were injected into the cortical tissue. This issue is significant since multiple features of the population response could simultaneously change during electrical stimulation, such as neurons’ firing rates or the correlations between cells, including the way in which neural signals are integrated over the entire population.

To address the limitations of previous causal manipulations of neuronal activity, we used an optogenetic approach to examine how the causally-evoked neural activity interacts with the endogenous activity across cell populations to influence perception. Specifically, we asked how the signals generated by a local neural population in V1 containing neurons with overlapping receptive fields, but heterogeneous orientation preference, are combined during a perceptual task. We answered this question by simultaneously activating two sub-populations of cells using a combination of visual and optogenetic stimulation. The populations were located at various functional distances, preferring similar or dissimilar stimulus orientations, but both carried information about the same visuo-spatial location. This approach takes advantage of the orderly representation of stimulus features, e.g., orientation, in primate V1, as a function of cortical distance^27,28^, hence similarly tuned populations are more likely to be physically proximal than dissimilarly tuned ones. We used a detection task for which the orientation-specific information signaled by the neural populations is irrelevant for task performance. The optimal strategy would be to pool all available information across all neurons. However, this does not appear to be the way the brain performs this operation. We demonstrate that neuronal signals are pooled across subpopulations of cells that are similarly tuned, within 45°, which corresponds to a lateral span of a few hundred microns, less than half the width of a hypercolumn. Beyond this threshold, neural populations appear to be treated independently, with perception being based on the population with the largest aggregate activity. We further show that whether the subthreshold, optogenetically-induced signal is integrated or not with the stimulus-driven activity is reflected in the local changes in the correlation structure across the population. Finally, we demonstrate that a network model based on computing the signal to noise ratio of neural populations at various functional distances (in the orientation domain) predicts perceptual performance on the detection task.

## Results

### Optogenetic stimulation locally activates cell populations in V1

Our goal was to selectively activate and record the activity of a localized population of glutamatergic neurons in V1, and subsequently test how the optogenetically injected signal is used for visual detection. We rendered populations of V1 glutamatergic neurons sensitive to light by expressing Channelrhodopsin-2 (ChR2), a blue light-sensitive cation channel, under the control of an *α-CaMKII* promoter. The ChR2 gene was delivered via columnar injections of a VSV-pseudotyped lentivirus carrying the ChR2-GFP gene (Fig. 1b), a construct that expresses exclusively in glutamatergic neurons in primate cortex^29^. Next, we coupled a fiber optic, for light delivery, to a 16-channel linear array, with the expressed goal of minimizing the distance between the two devices (range was 0–300 μm, see Methods for additional details). We recorded a total of 1031 units from two monkeys (both single unit and multiunit activity was included, henceforth called “units”). Of the total units, 597 (57.9%) showed a statistically significant response to light (comparing firing rates between laser on/off trials, see Methods), with 92% showing an increase in firing rate, and 8% showing a decrease. Examples of single unit responses to optogenetic stimulation are shown in Fig. 1c–e. Of the cells activated by light, 72.5% showed statistically significant responses to visual stimuli presented over their receptive fields (76% of the cells suppressed by light were also visually responsive).

We next measured the distance over which light modulates neural activity by counting the number of electrode channels (equally spaced 100 μm apart) over which we observed direct optogenetic stimulation (occurring ≤ 2 ms after light onset, Fig. 1f). Across the subset of sessions examined (*n* = 20), the average direct laser-induced activity spanned ~190 µm (full width at half maximum, aligned across sessions to channel with strongest laser response, Fig. 1g). The light-induced responses decayed to zero at distances greater than ~400 µm from the channel with the greatest activation. Light emitted from the tip of an optical fiber disperses through cortical tissue in an approximately spherical manner^30^. Assuming that the lateral spread of light is equivalent to vertical spread, if direct light-driven responses occur within a radius of 95–400 µm of the optical fiber, this extrapolates to a spherical volume of about 0.11–0.27 mm^3^ where light could activate transfected neurons. Weaker, indirect, network-based activation profiles (occurring at latencies ≥ 3 ms) were also present and spanned farther distances (Supplementary Fig. 1). These results provide evidence that optogenetic stimulation influences the responses of a small, spatially restricted subpopulation of neurons.

### Optogenetic stimulation influences behavioral performance

**Fig. 2:**
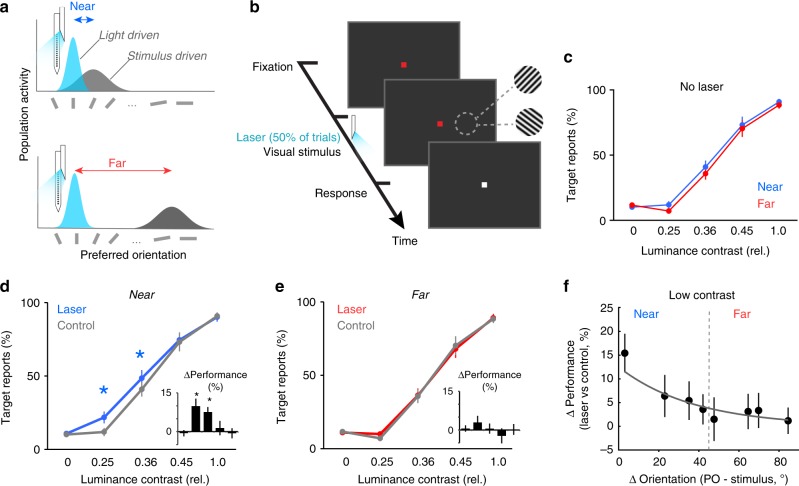
Stimulus detection performance increases when the stimulated neural populations are nearby. **a** Cartoon showing how two neural populations can be simultaneously activated using light and visual stimulation. The functional distance between the two populations is controlled by changing the orientation of the visual stimulus to be “near” (upper) or “far” (lower) from the preferred orientation of the light-driven population. **b** Detection task design. Following a fixation period, oriented gratings are presented at four different contrasts. Half of trials contain no visual stimulus, and half of the trials are paired with optical stimulation. All contrasts and orientations are randomly interleaved. Monkeys are cued to report the presence or absence of a stimulus. **c** In the control (no laser) condition, detection performance for “near” and “far” stimulus orientations is similar for all contrast levels (*P* > 0.50 all contrasts, Wilcoxon signed rank tests). Error bars show s.e.m. **d**, **e** Percent target reports across sessions. Optogenetic stimulation-induced change in behavioral performance when the neural population is exposed to the “near” (**d**) and “far” stimulus (**e**). Optogenetic stimulation improves the detection of the two lowest contrast stimuli in the “near” condition (*P = 0.0025, Kruskal–Wallis test, df = 4, Chi-squared value = 16.43, post hoc Wilcoxon signed rank test), but had no impact in the “far” condition (**e**). Error bars show s.e.m. across sessions. **f** The light-induced enhancement in behavioral performance decays as the orientation difference between the two subpopulations increases. Black dots represent the mean change in target reports (laser vs. control) across sessions according to the orientation difference between the visual stimulus and the preferred orientation of the light-activated population (Sessions were grouped into 10° increments. Vertical dashed line shows the division between “near” and “far” categories. Fit is exponential. Error bars show s.e.m.

**Fig. 3:**
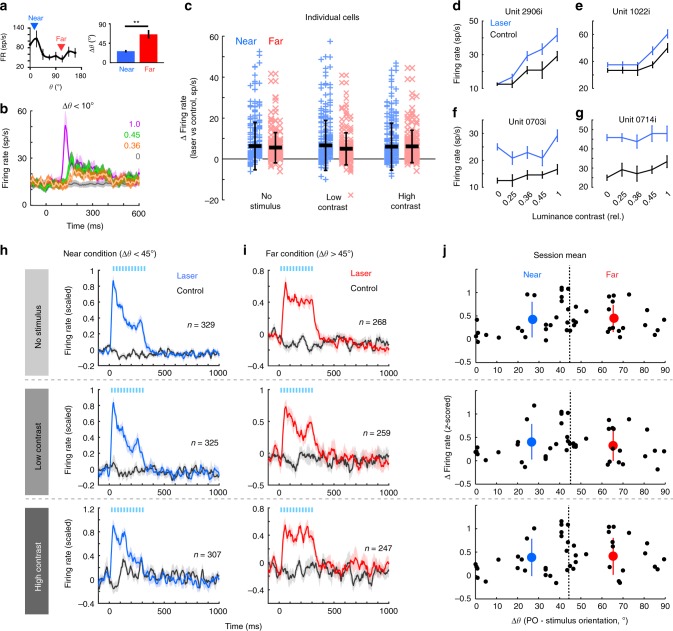
Light-evoked activity is uniform across stimulus orientations and contrasts. **a**, left Example orientation tuning curve for one neuron. Arrowheads represent the orientation of the “near” (blue) and “far” (red) stimuli presented in that session. **a**, right Mean difference between the neural population preferred orientation and stimulus orientation for “near” (blue) and “far” (red) conditions. **P = 2.8E-8, Wilcoxon rank sum test. Error shows s.e.m. **b** Mean firing rate of laser-responsive population on control trials, when the visual stimulus was within 10° of the preferred orientation of the neurons (*n* = 21). Colored lines represent different contrasts. Error bars show s.e.m. **c **Firing rate change associated with laser for individual units in “near” (blue) and “far” (red) conditions (first 300 ms following light/stimulus onset). Thick horizontal bars represent the mean. Error bars represent the s.d. No group is significantly different from another (*P* = 0.10, one-way ANOVA, df = 5, F-statistic = 1.86). **d**–**g** Contrast response functions for four example, light-responsive neurons (firing rates from 60–300 ms after stimulus onset). Error shows s.e.m. **h**, **i** Population mean firing rate (±s.e.m.) for all laser-responsive units recorded in “near” (**h**) and “far” (**i**) conditions. Rows correspond to contrast conditions. **j** Mean firing rate change per session across contrast conditions (rows). Black circles represent the mean firing rate changes across all simultaneously recorded light-responsive cells. Dashed vertical line shows the cutoff between “near” and “far” conditions. Large colored circles represent the mean change in firing rate across all “near” (blue) and “far” (red) sessions. Error bars show the standard deviation. The differences in laser-evoked firing rates are not statistically significant across stimulus contrast conditions (*P* = 0.35, Kruskal–Wallis test)

We examined how cortical signals are integrated during perception by simultaneously activating two nearby or distant neural populations using visual and optogenetic stimulation (Fig. 2a). Animals performed a contrast detection task in which they reported the presence or absence of a stimulus regardless of its orientation (Fig. 2b, see Methods). Stimuli of varying contrasts were present on 50% of trials. Optogenetic stimulation was also present on 50% of trials. To maximize the potential impact of the light-evoked spikes, we chose stimulus contrasts that would minimally drive neurons, while providing clear psychometric results in each animal. Stimuli consisted of luminance-varying contrast gratings, 2–3 deg in diameter, displayed for 800 or 1300 ms on a dark screen, randomly interleaved with ‘catch’ trials (no stimulus). Laser pulses were synchronized and limited to the onset of the visual stimulus. Across sessions, the mean time period over which laser pulses were delivered was 315 ms ± 18 s.e.m. (*n* = 56); individual laser pulses lasted 10 ms and were delivered at 35 Hz in 85% of sessions (range was 15–50 Hz; data was combined since we did not find significant differences in neuronal and behavioral responses, Supplementary Fig. 2). Extracellular recordings were always performed at the site of light stimulation. Within 1 mm of cortex, neurons share overlapping receptive fields, but exhibit systematic changes in tuning preference^4^ as a function of cortical distance^27,28^. Stimulus orientation was adjusted to be either similar (“near” condition, Fig. 2a upper) or dissimilar (“far” condition, Fig. 2a lower) to the mean preferred orientation (PO) of recorded, light-driven neurons, with the cutoff criteria between conditions set to 45°. Figure 3a (left) shows a typical tuning curve for one example unit illustrating “near” stimuli presented close to the peak of the tuning curve, and “far” stimuli presented at an orthogonal orientation. Across sessions (Fig. 3a, right), the mean “near” orientation difference (Δθ) between the population PO and stimulus orientation was 26.6° ± 2.9° s.e.m. (29 sessions), while the mean Δθ for the “far” condition was 65.6° ± 2.7° s.e.m. (27 sessions). Orientation preferences were significantly different between “near” and “far” conditions (*P* = 2.82 10^−8^, Wilcoxon ranked sum test). Neural responses of laser-responsive cells to optimally tuned stimuli (within 10° of the preferred orientation) are shown in Fig. 3b, and demonstrate that the virus injection did not noticeably alter stimulus response properties of transfected neurons.

Our experimental procedure allowed us to test whether the functional distance, i.e., difference in preferred orientation between the two activated subpopulations, representing the same patch of visual space, impacts how the elicited spikes are integrated to guide perception. That is, if all the spikes are integrated regardless of the orientation distance over which they occurred, optogenetic stimulation should influence perceptual reports in both “near” and “far” conditions. However, if only the spikes within restricted populations can be integrated, optogenetic stimulation would be expected to impact perception when paired with visual stimulation of the “near”, but not “far”, population. Since the orientation information was irrelevant to the detection task, the optimal strategy that maximizes reward would be to integrate across both populations in all conditions. As expected, in control trials (no laser) behavioral performance was independent of stimulus orientation, i.e., animals performed equally well for “near” and “far” stimuli (Fig. 2c; *P* > 0.50, Wilcoxon ranked sum test per contrast). Optogenetic stimulation, however, significantly increased the number of correct responses when laser and visual stimulation drove overlapping neural populations in the “near” condition, but had no impact on detection performance when the two populations were > 45° apart (“far” condition).

In the “near” condition (Fig. 2d) detection performance was improved by 9.9% ± 2.9 s.e.m. for the 0.25 contrast, and 7.6% ± 1.9 s.e.m. for the 0.36 contrast (*n* = 29 sessions, *P* = 0.0025 Kruskal–Wallis test, df = 4, Chi-sq value = 16.43; post hoc Wilcoxon signed rank test). Importantly, there was no significant difference in detection performance for the “far” condition on laser versus control trials (Fig. 2e, *P* = 0.63, Kruskal–Wallis test, df = 4, Chi-sq value = 2.56, *n* = 27 sessions). These results were consistent for individual monkeys, with both showing improved detection on laser trials at low contrasts in the “near” condition (M1: 7.7% ± 1.8 s.e.m., M2, 10.7% ± 3.5 s.e.m.; *P* *=* 0.0018, Kruskal–Wallis test, df = 5, Chi-sq = 19.2, post hoc Tukey–Kramer test), and no change in performance for either the no stimulus or high contrast conditions (*P* > 0.33, Wilcoxon signed rank test). Further, there was no significant difference in detection performance at low contrasts between monkeys (*P* = 0.20 “near” condition, *P* = 0.22 “far” condition, Wilcoxon ranked sum tests). This difference in detection performance in “near” versus “far” conditions was further confirmed within individual sessions, in which multiple stimulus orientations were used (see Methods for details). Behavioral performance systematically decreased as the orientation distance between the laser-driven and visually driven populations was increased (Fig. 2f). These results indicate that the signals from similarly tuned neural populations, within ~45° can be integrated to improve perceptual performance. In contrast, the signals generated by more differently tuned populations act independently. Additional control experiments (Supplementary Figs. 3–4) ensured that the behavioral effects reported here were not due to phosphene induction or laser-induced local heating.

### Optogenetic stimulation uniformly increases V1 activity

To investigate the neural basis of the behavioral improvement following optogenetic stimulation, we first examined the laser-driven changes in firing rates for all light-responsive units (*n* = 597 units, with each session contributing an average of 14.2 ± 1.5 s.e.m. light-responsive units). For individual units we quantified the light-evoked changes in firing rates during light stimulation (0–335 ms from laser onset, compared to control) across stimulus contrast and “near”/“far” conditions (Fig. 3c). Since behavioral and neural changes were similar for the two lowest (0.25 and 0.36) and highest stimulus (0.45 and 1) contrasts, results were grouped into “low” or “high” contrasts. The distributions of light-driven responses of individual units were not statistically different across contrasts for “near” (*n* = 329 units) and “far” (*n* = 268 units) stimulus conditions (Fig. 3c, *P* = 0.10, one-way ANOVA, df = 5, F-statistic = 1.86). In all cases, laser stimulation increased firing rates significantly above control level (*P* < 0.01, Wilcoxon signed rank test, all conditions). Distributions of all firing rates are shown in Supplementary Fig. 5. Examining individual neuron responses revealed differences in light-evoked activity as a function of stimulus contrast (Fig. 3d–g shows contrast responses for four units). Interestingly, many neurons showed weak modulation by the visual stimuli, but strong modulation by the light (Fig. 3f, g). Population responses of all light-responsive neurons across conditions are shown in Fig. 3h, i (see also Supplementary Fig. 6 for population responses including all stimulus-responsive neurons, regardless of light responsiveness from “near” sessions). All light-responsive neurons were included in subsequent analyses, unless otherwise stated.

Since the light-induced increase in behavioral performance decreases as a function of orientation difference (Fig. 2f), we asked whether there is a systematic change in firing rates for the mean population activity in the “near” and “far” conditions. We organized sessions based on the difference between the mean PO of the population and stimulus orientation (Δθ, Fig. 3j), and averaged the z-scored responses of all simultaneously recorded units in a session. However, we found no systematic fluctuation in the laser-evoked changes in population firing rate across stimulus orientation or contrast conditions (*P* = 0.35, Kruskal–Wallis test). Since our recordings were perpendicular to the cortical surface we expected very similar tuning preferences within the recorded column. However, in V1, the smoothly varying iso-orientation domains are punctuated with so-called “pinwheel centers”, in which orientation preferences of nearby columns changes rapidly over very short distances^31^. To quantify whether any of our recordings included sites near possible pinwheels and to examine the effects of tuning heterogeneity on laser-evoked behavioral changes, we calculated the tuning variance on individual sessions. This analysis confirmed that the vast majority of sessions were recorded in iso-orientation domains, with very similar tuning across the population (Supplementary Fig. 7a–b). Across “near” and “far” sessions, the small variability that was present was not well correlated with the change in behavior (Supplementary Fig. 7a–b). Altogether, these findings demonstrate that the difference in behavioral performance between the “near” and “far” conditions cannot be sufficiently explained by differences in the light-driven neuronal activity of individual neurons. This led us to examine whether the difference in behavioral performance could be explained by differences in the propensity of the local network to integrate information from the light-driven population across “near” and “far” conditions, which might be detectable using population-scale metrics.

### Optical stimulation differentially affects correlations

**Fig. 4:**
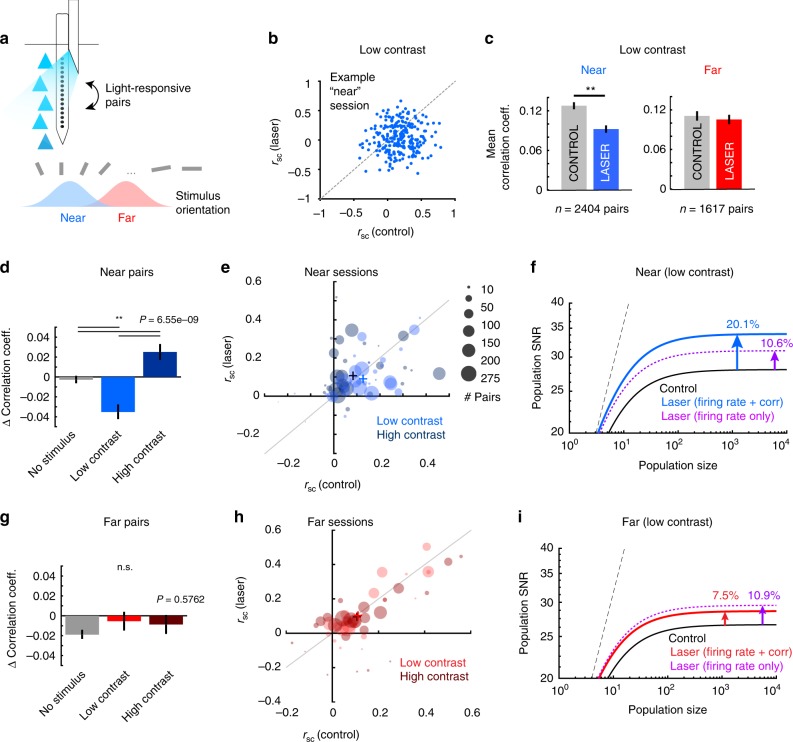
Optogenetic stimulation alters noise correlations and network performance in the “near” condition. **a** Noise correlations between light-responsive pairs along laminar electrode were measured across stimulus conditions. **b** Changes in noise correlations (“*r*_sc_”) for one example “near” condition session, during low contrast stimulus presentation. Each point represents correlation coefficients for one pair of laser-responsive cells in control and laser trials. **c** Population mean noise correlations for laser-responsive cell pairs during control (gray bars) and laser trials (colored bars) for “near” (left panel, blue) and “far” (right panel, red) conditions during low contrast presentation. (**Near sessions: *P* = 2.85e-6, Wilcoxon signed rank test, z-statistic = −4.68. Far sessions: *P* = 0.25, Wilcoxon signed rank test, z-statistic = −1.14). Error bars represent s.e.m. **d**, **g** Mean change in noise correlations following optogenetic stimulation for “near” (**d**) and “far” (**g**) across all stimulus conditions. Kruskal–Wallis test. Error bars are s.e.m. **e**, **h** Mean change in noise correlations for individual “near” (**e**) and “far” (**h**) sessions, at low (light circles) and high (dark circles) contrasts. Plus signs indicate cross session pair mean in each condition. Area of circle is proportional to the number of pairs from each session. **f**, **i** Impact of noise correlation changes on population SNR. Plots show SNR for hypothetical populations of increasing size for “near” (**f**) and “far” (**i**) subpopulations during presentation of low contrast stimuli. Solid lines show the SNR values during the control (black), and laser conditions for “near” (blue, **f**) and “far” (red, **i**) activated populations. Dotted magenta lines represent the change in SNR attributable to light-induced increase in firing rates alone, if noise correlations remain unchanged. Dashed black line represents the hypothetical SNR if there was no correlated noise

Correlated variability (noise correlations) between neurons is believed to impact the information encoded in population activity and limit the benefits of pooling across populations of neurons^18,32–34^. To test whether optogenetic stimulation reveals differences in information encoding, we calculated noise correlations (trial-by-trial covariation in spike counts) between pairs of simultaneously recorded light-responsive neurons (Fig. 4a). We focused on the stimulus condition associated with improved detection performance, and combined the z-scored data from the two lowest contrasts to increase estimation accuracy. Optogenetic stimulation reduced correlations in the “near” condition (28% reduction, Fig. 4b–e, *P* = 2.85e-6, Wilcoxon signed rank test), but had no impact in the “far” condition (Fig. 4c, g, h *P* = 0.25, Wilcoxon signed rank test. See Supplementary Fig. 8 for correlation coefficients in other conditions). Further, we found this “near” reduction in correlations was more prevalent in sessions with more homogeneously tuned units (Supplementary Fig. 7c). Note that noise correlations changed in the opposite direction as firing rates during laser stimulation in conjunction with low contrast stimuli. While concurrent optogenetic and visual stimulation with low contrasts reduced correlations (Fig. 4c–e, laser minus control mean correlation difference was −0.035 ± 0.053 standard deviation), the presence of a high contrast stimulus resulted in an increase in light-evoked correlations (mean change 0.024 ± 0.056 s.d.; *P* = 6.84e-4, Wilcoxon signed rank test). In the absence of a stimulus, correlations were unchanged (Fig. 4d, mean laser vs. control change −0.009 ± 0.17 s.d.; all distributions were different from each another, *P* = 6.56e-9, Kruskal–Wallis test, df = 2, Chi-square value = 37.69, post hoc Tukey test). By contrast, in the “far” condition, optogenetic stimulation had no effect on pairwise correlations regardless of stimulus contrast (Fig. 4g, h, *P* = 0.58, Kruskal–Wallis test, df = 2, Chi-squared value = 1.1). The dependence of the noise correlation changes on (1) the orientation distance between visual and light-driven neural populations (Fig. 4c), (2) the stimulus contrast (Fig. 4d, e), and (3) the tuning similarity of the cell pairs (Supplementary Fig. 7c), strongly indicate that the observed changes in noise correlations are not due solely to the light stimulation, but rather, are reflective of the underlying complex interaction between the visually driven and optically driven neural populations in perceptually relevant conditions.

Correlations have been hypothesized to limit the benefits of pooling across populations of neurons by imposing an upper asymptotic limit on the signal-to-noise ratio^18^, a measure of the fidelity of signal transmission and detection by neurons and synapses^35^. To estimate how the light-induced changes in correlations impact network stimulus coding, we calculated population SNR for different sizes of a hypothetical neural ensemble that shares the same statistical features as our recorded populations^18^. We thus quantified the impact of the combined laser-induced increase in firing rates and decrease in correlations on SNR, separately for the “near” (Fig. 4f) and “far” conditions (Fig. 4i). In the “near” condition, the laser-induced changes in firing rates and reduction in correlations led to a 20.1% increase in the asymptotic SNR compared to no-laser condition (Fig. 4f, solid blue line). To assess the contribution of changes in firing rate alone, we recalculated SNR by ignoring the changes in correlated variability (assuming that optogenetic stimulation leaves correlations unchanged relative to control). In the “near” condition, firing rate changes alone were associated with only a 10.6% increase in population SNR (Fig. 4f, dotted purple line), which was comparable to changes observed in the “far” condition (Fig. 4i, dotted purple line). Similarly, in the no-stimulus condition, the firing rate increase in the absence of changes in correlations led to a small increase in population SNR (9.8% compared to the no-laser condition).

### Neural signal pooling model captures behavioral performance

**Fig. 5:**
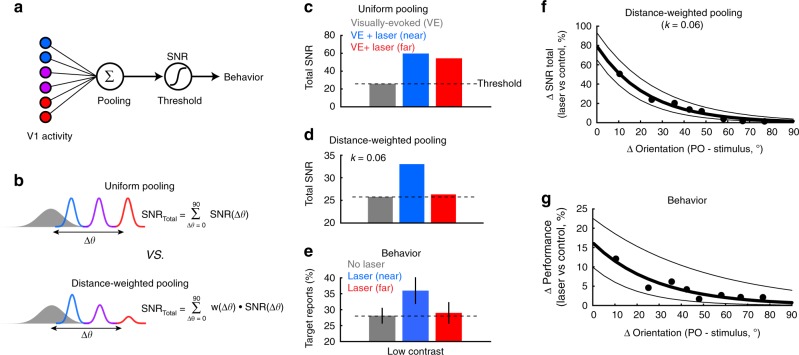
Functional distance-weighted pooling correlates best with detection performance. **a** Basic model of sensory detection relying on pooling signals across neural populations. Stimulus detection requires the pooled signals from V1 (colors represent different tuning preferences) to exceed a critical SNR threshold. **b** Two possible models of signal pooling. Upper A uniform pooling rule in which the total SNR is based equally on the sum of the visually driven response (gray, filled distribution) and the laser-driven response (colored, unfilled distributions), regardless of tuning difference (colors). Lower A distance-weighted pooling rule, in which the contribution of the laser-driven population is weighted by its orientation-difference from the visually driven population. The weight (w) is a simple exponential decay (see main text). **c** Total SNR predicted by uniform pooling for low contrast stimuli. The laser-driven neuronal activation in both the “near” (blue) and “far” (red) conditions substantially increases total SNR above the control condition (gray). Dashed black horizontal line estimates the global SNR threshold based on the control condition. **d** Total SNR predicted by functional distance (difference in orientation) weighted pooling for low contrast stimuli. Only the laser-driven neuronal activation in the “near” condition (blue) increases the total SNR substantially above the control condition, matching well with the laser-induced change in behavior (**e**). **e** Behavioral detection performance for low contrast stimuli in the absence of laser stimulation (gray), and with laser stimulation in the “near” (blue), and “far” (red) conditions. Error bars represent s.e.m. across sessions. Dashed black horizontal line estimates the detection threshold based on the control condition. **f** Percent change in total SNR following laser stimulation using functional distance-weighted pooling, calculated as a function of the orientation difference between the laser and visually driven populations. SNRs were estimated from bootstrapped data sampled every 10 sessions (ordered by Δθ), sliding every five sessions. Thick black line shows an exponential fit to the data points. Flanking thin black lines show the 95% confidence intervals for the fit. **g** Change in behavioral performance (laser vs. control), averaged across the same sessions used in **f**. Thick black bar shows an exponential fit to the data. Flanking thin black lines show the 95% confidence intervals for the fit

SNR quantifies the changes in encoded information in the laser-driven subpopulations in the “near” and “far” conditions. However, to examine how the information encoded in the laser-driven population is combined with that in the visually driven population, we explored pooling rules that would best account for the observed changes in behavior. Population SNR has been previously used as a measure of detectability performance^18,36^ under a pooling model assuming that spikes from all the cells in the pool are integrated without reference to their origin^37^. In brief, if the sum of the pooled signals exceeds a set threshold, it leads to a sensory percept (Fig. 5a). To integrate signals arising from the populations of cells driven by the visual stimulus and by optogenetic stimulation, SNRs from the two groups were combined according to two possible schemes (Fig. 5b): (i) Pooling that samples uniformly across all active subpopulations regardless of orientation distance (“Uniform pooling”; see also Supplementary Fig. 9b, c for a related variation of this rule), and (ii) pooling that samples in an orientation distance-dependent manner according to the orientation preference difference between subpopulations (“Distance-weighted pooling”).

Uniform pooling across a small cortical area can be described as a linear summation of the relevant neural signals regardless of functional distance. We thus combined the SNRs associated with the laser-driven and visually driven subpopulations (Fig. 5c), and set the stimulus detectability threshold (Fig. 5a) as the total SNR in control associated with the low contrast stimuli (Fig. 5c, dashed horizontal line). The uniform pooling scheme yielded a robust increase in total SNR well above the detectability threshold in both near and far conditions, which is inconsistent with the laser-induced changes in behavioral performance (Fig. 5e). We next examined an orientation distance-weighted pooling model (Fig. 5b, d), in which the contribution of the laser-driven population is scaled depending on the orientation distance to the visually driven population. Our model assumes that the responses of the recorded neurons on control trials are representative of the larger stimulus-responsive neural population, and focuses on the responses within the first 300 ms following stimulus and/or laser onset. The scaling weight (*w*) is given by *w*(Δθ) *=* e^−*k*(Δθ)^, where *k* is a constant that determines the rate decay of pooling, and Δθ is the orientation difference between the visually-driven and laser-driven populations. When *k* = 1, this model is equivalent to uniform pooling, while *k* = 0 represents independent populations (no pooling). We varied *k* (Supplementary Fig. 9a) to find the best fit to behavioral results in the near and far conditions. At *k* = 0.06, the distance-weighted pooling of SNR (Fig. 5d) was consistent with the observed changes in behavior (Fig. 5e). An exponential weight was chosen due to the observed non-linearity in behavioral performance (Fig. 2f). When stimuli were presented at high contrast, total control SNR exceeded the lower limit of the detection threshold (Supplementary Fig. 9c–e). Therefore, the additional laser-driven activity is not expected to modulate behavior (Supplementary Fig. 9b–f). Furthermore, by gradually varying the orientation difference (Δθ) between the two subpopulations we found that the distance-weighted pooling rule provides good agreement between the laser-induced changes in total SNR and animal’s detection performance (Fig. 5f, g, *R* = 0.88, *P* = 0.0038 Pearson correlation).

## Discussion

Our study used optogenetics to activate excitatory neurons in macaque V1 in order to identify rules of perceptually relevant signal integration. We demonstrate that neuronal population activity is pooled in a functional distance-dependent manner, in accordance with the spatial organization of orientation in V1. We propose that perceptually relevant information is pooled across functionally-confined local cortical populations in V1, and that this pooled population vector is further transmitted to downstream areas to guide behavior. Furthermore, we identified unique changes in the local correlation structure that manifest only when the population signals are integrated perceptually.

Signal pooling has been previously examined at the single neuron level in the context of spatial summation, whereby neural activity in V1 becomes stronger in response to stimuli of increasing size due to a stronger drive provided by geniculate feedforward projections to V1^38^. As stimuli continue to increase in size modulatory surround networks are recruited and dampen stimulus responses, known as surround suppression^39^. While previous surround suppression studies have contributed to our understanding of underlying network principles responsible for shaping V1 responses, they have not addressed how such signals are pooled to drive perceptual reports.

The functional architecture of macaque V1 has been suggested to play a role in perceptual judgments^40^, but this idea has not been empirically tested. Several studies using electrical and/or visual stimulation have suggested that neurons in striate and extrastriate cortical areas may contribute to perception based on their tuning to features that are task irrelevant, but spatially organized^24,41–43^. For example, medial temporal neurons (MT) in macaques are organized topographically according to motion direction and binocular disparity. In a motion discrimination task, electrical microstimulation of neurons tuned to both features does not affect behavioral performance unless the test stimulus is also presented at the cells’ preferred disparity plane^44^. Similarly, choice-related activity in V1 populations has been reported^45^ for stimulus features that have a systematic topographical organization (e.g. orientation), but not for stimulus features that lack such organization (e.g., disparity). These findings are consistent with our conjecture that perceptual decisions are based on the cumulative activity of spatially/functionally clustered sensory cortical subpopulations.

Probability summation has been the gold standard for modeling behavioral performance in sensory detection tasks^35,46^. For instance, in the max operator model, the responses of multiple independent channels are monitored and if any response exceeds a threshold, the choice is determined based on the response of that individual channel. Thus, the likelihood of a correct detection increases proportionally to the number of channels, e.g., two different stimuli simultaneously presented are more likely to be detected than either alone. Our findings argue that probability summation models cannot adequately explain signal integration relevant for perception. Namely, the decision-making process does not query the activity of individual neurons (i.e., “channels” in the model do not represent individual cells), otherwise optogenetic stimulation would increase stimulus reports across all conditions. Alternatively, signals are summed first across separate neural populations, and the max operator acts upon such population signals^47^. In this framework, the strong light-driven activity can be quenched when it is pooled within a larger, otherwise silent population (i.e., the “far” condition) and thus cannot contribute to perception. Importantly, our results suggest a possible kernel size for this signal pooling area encompassing neurons tuned within 45°, which corresponds to approximately several hundred microns of cortical space in macaque V1^28^.

One key observation in our study is that noise correlation structure is changed upon optogenetic stimulation only in the “near” condition, where noise correlations decline in the presence of low-contrast stimuli, but increase in conjunction with higher contrast stimuli (Fig. 4d). The structure of noise correlations has previously been shown to be both stimulus^48–50^ and task dependent^51^. As correlations were unchanged during optogenetic stimulation alone (no stimulus) or in the “far” condition, these observations reveal a differential engagement of the local network when the visual stimulus is near the preferred orientation of the population. This hidden network state can be revealed by optogenetic stimulation, which then shifts the local network from one state of correlations to another^34,52,53^. This shift in correlations could be potentially explained by changes in the relative strength of local excitation and inhibition. When low contrast stimuli are presented, responses are weak, and thus the local strength of inhibition is also weak. In this condition, optogenetic stimulation supplies additional drive to excitatory cells, whose responses are closely tracked by local inhibitory cells and act to reduce correlations between cell pairs^34,52^, similar to proposed mechanisms for reduced noise correlations during spatial attention^54,55^. However, when higher contrast stimuli are presented, it is conceivable that a larger local network is activated leading to an increase in recurrent excitation, which could act to amplify variability^56^.

Several computational models have been proposed to explain the sources of correlated variability^49,56,57^. Our results are most consistent with a recent model^57^ suggesting that correlated variability can be suppressed or facilitated depending on whether the coupling between neurons is excitatory or inhibitory. This model proposes that correlations are reduced at low contrast and increased with higher contrast when center and surround stimuli have similar directional biases. This is the case in our study, as stimuli were larger than classical V1 receptive fields and likely engaged near-surround^58^ circuits. Thus, known local connectivity provides a potential mechanism to explain why optogenetic stimulation reduces correlations in conjunction with low contrast stimuli, but increases them with higher contrast stimuli. However, our study used luminance-varying contrasts, with very low overall mean luminance. Neural contrast responses are known to change as a function of luminance^59^. Our luminance levels were very low across all contrasts, and are unlikely to have significantly altered V1 contrast responses, but further experiments are required to parametrically evaluate at which luminance and stimulus contrast levels the network switches from one operating regime (increased drive decreases variability) to another (increased drive increases variability).

Our results indicate that optogenetic stimulation alone is unable to drive a percept. This is contrary to electrical microstimulation studies in humans and non-human primates^23,60^ which clearly demonstrate that stimulating V1 evokes visual percepts at the receptive field locations of the activated neurons. The paucity of studies^61–63^ showing clear behavioral modifications utilizing optogenetic stimulation in non-human primates hint that optogenetic stimulation provides a much weaker drive to the network than electrical microstimulation, which has been demonstrated by direct side-by-side comparisons with electrical microstimulation^62,64^, by spatial measurements of optogenetic activation using fMRI^64,65^, and by intrinsic imaging^66^. This is consistent with the idea that perceptual decisions are based on the pooled activity of many neurons and, in order to influence a percept, a sufficiently large cell assembly must be targeted.

As the tools for non-human primate optogenetics continue to expand^67^, dissecting the neural circuitry for sensory perception and decision making at single-cell resolution is increasingly probable. Our study demonstrates that optogenetics can be used to probe the state of neural networks that would otherwise remain invisible. The similarity of microcircuitry across the cortex suggests that the pooling rule proposed here may constitute a general coding strategy utilized in sensory modalities beyond vision.

## Methods

### Ethics statement

All experiments were performed in accordance with protocols approved by the Animal Welfare Committee (AWC) and the Institutional Animal Care and Use Committee (IACUC) for the University of Texas Health Science Center at Houston (UTHealth).

### Animals and surgery

Two male rhesus monkeys (*Macaca mulatta*; M1, 8-years-old, 15 kg; M2, 12 years old, 13 kg) were used in the experiments. Monkeys were previously trained in discrimination/detection tasks and were surgically implanted with a titanium headpost device and two 19 mm recording chambers (Crist Instruments) in areas V1 and V4.

### Viral vector injections

ChR2 was expressed specifically in V1 excitatory cells using the same lentiviral vector as used previously in monkeys by Han et al.^29^. High titer (>10^9^ IU ml^−1^) purified lentivirus was obtained from the University of North Carolina Gene Therapy Center Vector Core. The virus was injected through a 29 gauge needle connected via mineral oil filled tubing to a Hamilton syringe mounted on a perfusion pump (KD Scientific). The needle was advanced by a precision, computer controlled micro-manipulator (NAN instruments) to a pre-established depth (corresponding to the lowest depth at which unit activity was found in preliminary experiments). After a 15 min of waiting (to allow for stabilization), 1 µl of virus suspension was delivered over a 10 min period. The needle was then retracted slowly upward (0.1 mm min^−1^) in 200–300 µm steps and an additional 1 µl of virus suspension was delivered at 3–4 additional depths. Five minute wait periods were interleaved before and after each virus delivery and retraction steps. Multiple injections were performed in each V1 chamber (8 for M1, 11 for M2) closely grouped together and forming a rectangular pattern across the cortical surface.

### Behavioral paradigm

During each recording session, monkeys performed fixation tasks to identify light-responsive units, map receptive field locations and determine the preferred orientation of units. Monkeys sat in conventional primate chairs, head-restrained, in front of a computer monitor 90 cm away. Eye position was continuously monitored using an infrared, mirror-based eye tracking system operating at 1 KHz (EyeLink II, SR Research Ltd.). Monkeys maintained fixation on a 0.4 deg central square for a period of time in order to obtain a juice reward. Receptive field locations were mapped using 0.33 deg reverse correlation stimuli (full contrast, sinusoidal gratings, four orientations) presented at random screen locations. To initially identify light-responsive units, monkeys performed a fixation task with laser stimulation (24 Hz, 10 cycles, 10 ms width). Orientation preference was determined as described in detail below (Orientation selectivity).

For the principle experiments, monkeys performed a detection task using gray-scale sinusoidal gratings of various luminance-varying contrasts. Stimuli were generated using Matlab with Psychophysics Toolbox^68^ and presented binocularly on a computer screen on a dark background. Monkeys fixated on a central point (0.4 deg in size) within a 1 deg fixation window while stimuli with a diameter of 2–3 deg were displayed at 2–4 deg eccentricity. The location and size of the stimuli covered the multiple receptive fields of the cells recorded. Stimuli had a fixed spatial frequency (1.7 cycles per degree), displayed for 1300 ms (when only one orientation was present) or 800 ms (when multiple orientations were used), starting 450–1000 ms after fixation onset. The orientation of the grating stimuli could vary both within and across sessions. Stimulus orientations were first chosen based on the coarse, online estimates of the neural population preferred orientation that was later precisely measured offline following spike sorting. The final “near” or “far” categories were applied after this step.

A subset of sessions (*n* = 12) included both “near” and “far” orientations (multiple orientations condition, one orientation per trial, all trials randomly interleaved). For these sessions, behavioral performance was enhanced with laser stimulation only at low contrasts (0.25 and 0.36) in the “near” condition, but not in the “far” condition (*P* = 0.0053, Kruskal–Wallis test, df = 4, Chi-sq. value = 14.74; *P* = 0.026 post hoc Wilcoxon ranked sum test, comparing change in behavior with laser at low contrasts in “near” versus “far” conditions), hence confirming our main findings (Fig. 2d, e). The low luminance contrast values were chosen such that stimuli elicited small, unsaturated neural responses, around the psychophysical detection threshold determined for each monkey in preliminary experiments. Peak (mean) luminance measurements for stimulus contrasts labeled 0.25, 0.36, 0.45, 1.0 were 0.107 (0.0935), 0.120 (0.1), 0.133 (0.1065), 0.280 (0.18) cd m^−2^, respectively, while the minimum luminance and the no stimulus condition had a luminance of 0.08 cd m^−2^ (Tektronix, J17). In each experiment, stimuli could have one of 4 different luminance contrasts and were present on 50% of the trials. Monkeys were required to maintain fixation throughout each trial. If fixation was broken, trials would abort. Monkeys held a metal lever at the onset of each trial and maintained contact until the behavioral response was cued by the disappearance of the fixation point. At the end of stimulus presentation, monkeys were required to signal the presence of the stimulus by releasing the lever or maintaining contact if no stimulus was displayed. Correct behavioral responses were rewarded with 5 drops of juice. Optogenetic stimulation was triggered simultaneously with the onset of the visual stimuli (or at the time when a stimulus was expected, on no-stimulus trials). The optogenetic stimulation was aligned to the start of the visual stimulus in order to approximately coincide with the robust transient response of V1 neurons. This was chosen because there was a clear decay in the ability of the optical stimulation to drive V1 responses with each subsequent laser pulse (Fig. 1c–e). We also used a pulsed rather than continuous light protocol in order to avoid potential cell damage^30^. Optogenetic stimulation was present on 50% of trials, randomly and evenly distributed for each stimulus condition. Each session consisted of 160–720 total trials. Trials were split according to stimulus orientation, and then analyzed independently.

### Optogenetic stimulation and electrophysiology

Optical stimulation was achieved using a 100 mW, TTL controlled, DPSS blue (473 nm) laser (RGBLase) coupled to a 200 µm optical fiber. The end of the fiber was inserted into a 356 µm cannula and mounted on the NAN Microdrive. The light intensity at the tip of the fiber was within the range of 6.7–14.5 mW per mm^2^ (integrating sphere sensor, S124C Thor Labs). Light intensity was held constant across experiments. Prior to each experiment, both devices were lowered to the expected depth. We sought to minimize the distance between the optical fiber tip and the probe. Our custom grid allowed the devices, when parallel, to come within 300μm of each other, but we found that by manipulating the angle at which the devices entered the guide tubes, we could achieve even closer spacing at the target depth, with the shafts of the optical fiber and the electrode nearly touching (range was ~ 0–300 μm, with most sessions having a spacing of ~ 100–200 μm). The lateral distance between devices is based on measurements at the bench. There is no method by which to measure this distance inside the brain. Prior to each experiment, the fiber optic was aligned with the upper third of electrode contacts. The shafts of both devices were marked with registration lines that allowed us to independently confirm that the vertical alignment of the fibers was preserved once the dura was penetrated. Optical stimulation of the neurons was achieved by delivering 10–15 bursts of 5–15 ms light pulses at 15–50 Hz. The laser output was regulated via TTL pulses driven by a waveform generator (Model 3220 A, Agilent Technologies), controlled by the experiment control module (FHC Inc). Data across sessions was combined since there was no significant difference across stimulation frequencies tested (Supplementary Fig. 2). For the additional control sessions (Supplementary Fig. 4), recordings were performed utilizing the same procedures described above, except that the optic fiber was positioned 1–3 mm from the nearest injection site. No light-evoked neural activity, or behavioral modulation was observed.

The laminar electrodes (U-probe, Plexon Inc) consisted of a linear array of 16 equally spaced contacts (100 µm inter-contact spacing). Each electrode contact was 25 µm in diameter and platinum iridium coated. The impedance at each contact was 0.3–1.0 MΩ. Real-time extracellular neuronal signals (simultaneous 40 kHz A/D conversion on each channel) were analyzed using the Multichannel Acquisition Processor system (MAP system, 64 channel, Plexon Inc). Neural activity was amplified, filtered, viewed on an oscilloscope and heard through a speaker. Light-induced artifacts were sometimes present in the local field potentials, but not in the high-pass filtered spike data. This was confirmed with periodic recordings in saline.

### Phosphene controls

Electrical stimulation has long been known to induce artificial percepts known as phosphenes^23^. Non-cell-type-specific optogenetic stimulation has also been linked to phosphene induction^61^. Here, in order to test whether the optogenetic stimulation parameters used in these experiments is sufficiently strong to drive a phosphene, we examined four measures reflective of phosphene induction: (i) false alarm rates, when animals incorrectly reported the presence of a stimulus; incomplete trial counts due to (ii) fixation breaks or (iii) premature response bar releases, and (iv) microsaccade count. First, we examined the differences in false alarm rates (type 1 errors) between control and laser trials, when monkeys produce the behavioral response associated with the visual stimulus when, in fact, no visual stimulus is present. We found no significant difference in false alarm rates in any session type in which laser responses were recorded (Supplementary Fig. 3a, *P* = 0.45, Wilcoxon signed rank test). Second, we reasoned that the sudden appearance of a phosphene may be distracting to the animal and lead to reflexive shifts in attention that may result in erroneous eye movements or behavioral responses. Such breaks in fixation or premature behavioral responses would result in aborted trials. Again, we found no significant difference between laser and control trials in the number of aborted trials in each session due to fixation breaks (Supplementary Fig. 3b, *P* = 0.76, Wilcoxon signed rank test for all comparisons in this figure) and premature bar releases (Supplementary Fig. 3c, *P* = 0.12, Wilcoxon signed rank test). Lastly, we counted the number of microsaccades that occurred during optical stimulation and control trials in each session (Supplementary Fig. 3d), but the differences were not statistically significant across the two animals (*P* = 0.57, Wilcoxon signed rank test). We thus concluded that optogenetic stimulation under our experimental conditions is unlikely to have induced phosphenes.

### Cell classification

Spike sorting was performed offline using waveform-based principal component analysis software (Offline sorter, Plexon Inc). Subsequent analysis was performed using custom scripts (Matlab, Mathworks Inc). We identified cells based on their functional responses to the light stimulation and to the visual stimuli. Light sensitive cells were identified by comparing the firing rates on trials during the laser-on period (first 300 ms) with the equivalent period in the control trials in the absence of a visual stimulus (statistical criterion was *P* < 0.05, Wilcoxon rank sum test). Visually responsive cells were identified by comparing the firing rates calculated over 300 ms, beginning 35 ms after the onset of the visual stimulus, and the corresponding period during the no-stimulus trials. Visual responsiveness was assessed using responses to the oriented gratings present in the detection task and/or using the full contrast, multi-orientation stimulus used to map tuning preferences. This latter criteria was necessary for the “far” condition, when the cells’ preferred orientation was not represented by the visual stimulus used in the detection task, and hence would not be substantially driven by it. Only control trials (without light stimulation) were considered for stimulus responsiveness.

### Orientation selectivity

Orientation preference for each cell was measured before the behavioral task. Monkeys were required to fixate on the central fixation spot while a reverse correlation stimulus consisting of a sequence of 48 circular 100% contrast sinusoidal gratings (eight equidistant orientations randomly flashed at 30 Hz) was presented for a total duration of 1.6 s. The size and location of the stimuli were kept identical to the ones used in the detection task. Preferred orientation and orientation selectivity index (OSI) for each neuron were computed from Fourier components extracted from the orientation tuning curves as described previously^40,69^. To obtain the mean orientation preference for each penetration, we averaged over all responsive neurons within the vertical column spanned by each laminar electrode. In those sessions in which we identified laser responsive neurons on both laminar electrodes (5/15 sessions), we estimated the tuning of the entire population by averaging orientation preferences across both electrodes (the differences in preferred orientation across the electrodes was between 5–25°). For the remaining sessions in which we used 2 electrodes (10/15), laser-induced responses were found only along one electrode, and in this case the data from the non-light responsive electrode was not included in the analysis. Variance of tuning across the population of units in individual sessions (Supplementary Fig. 7) was computed using circular statistics (CircStat toolbox for MATLAB^70^).

### Noise correlations and signal to noise ratio (SNR) analysis

Noise correlations were calculated for pairs of simultaneously recorded laser-responsive neurons in each session using methods identical to Hansen et al.^34^, using the z-scores of spike counts obtained from the first 335 ms of each trial, separately for laser and control trials. Aberrant trials in which either of the cell pair’s firing rate was greater than 4 standard deviations from the mean were excluded, as were neurons whose mean firing rate across trials was <1 spikes per second. To compare across visual stimulus conditions given the similarity in firing rate and behavioral changes, the two lowest and the two highest stimuli were grouped together to increase the total number of trials for each pair, and thus increase the reliability of the noise correlation coefficient estimate. This was done by first z-scoring the firing rates in each contrast condition, then combining trials across stimuli prior to calculating the noise correlation coefficient.

Population SNR (SNR_p_) was calculated using methods identical to^18^ (equation 1), which, estimates the contribution of *M* identically distributed neurons to a sensory decision pool as a function of correlation strength between neurons.

1 $$SNRp = \frac{{{\mathrm{M}} < {\mathrm{X}} > }}{{\sqrt {M\sigma ^2 + M\left( {M - 1} \right)r\sigma ^2} }}$$

Where M is the number of neurons, < X > is the mean spike count for M neurons, σ^2^ is the standard deviation of this spike count, and *r* is the mean noise correlation. Calculations were performed with spike counts from laser-responsive neurons, separately for laser and control trials using the first 335 ms following stimulus onset. For Fig. 5f, g, to calculate the changes in total SNR we arranged the neural data according to the average difference in orientation of the simultaneously recorded cell population and that of the stimulus orientation in any one session, ranging from smallest to largest. We then calculated the total SNR based on average responses (firing rates and noise correlations) from bootstrapped samples from cells distributed across 10 sessions. This process was repeated sliding in increments of five sessions until all sessions were included. For each group of 10 sessions, we also calculated the average difference in orientation (plotted on the abscissa) and the average change in behavioral performance following optical stimulation (Fig. 5g).

### Statistical analyses

All implementations of the Kruskal–Wallis and ANOVA tests were one-way, while Wilcoxon signed rank, and rank sum tests were two-way.

### Microsaccade count

Eye positions for a 350 ms interval, starting 50 ms before laser onset, were convolved with a low-pass linear finite impulse response (FIR) filter with a 50 Hz cutoff frequency. Microsaccades were identified as deflections of the eye position during which eye velocity exceeded 10° per second for at least 10 consecutive ms, and eye acceleration exceeded 1000 degrees per second during a 40 ms interval centered at the maximum of the eye velocity. Successive microsaccades separated by <30 ms were considered a single eye movement.

### Reporting summary

Further information on research design is available in the Nature Research Reporting Summary linked to this article.

## Supplementary information

Supplementary Information

Reporting Summary

## Data Availability

The data upon which this study was based are available from the corresponding author upon reasonable request.

## Code availability

Data analysis was performed using MATLAB (Mathworks). The code upon which this study was based are available from the corresponding author upon reasonable request.
