# Supplementary material for: Integration of cortical population signals for visual perception
Source: Nat Commun. 2019 Aug 23;10:3832. doi: 10.1038/s41467-019-11736-2 (PMC6707195; doi:10.1038/s41467-019-11736-2)
Supplement: Supplementary file 1 — Supplementary Information [file 41467_2019_11736_MOESM1_ESM.pdf]

Integration of cortical population signals for visual perception

Andrei et al. 2019

Supplementary Figures

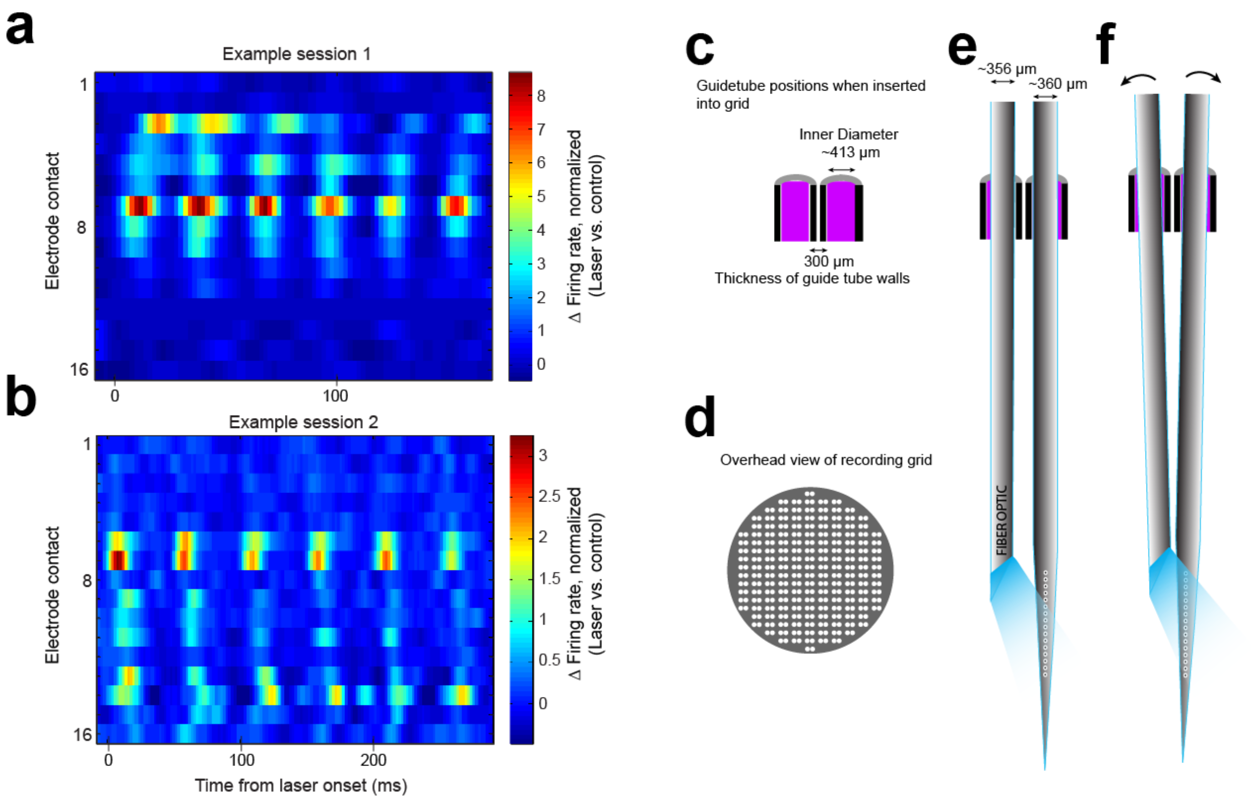

Supplementary Figure 1 Optically activating and recording from laminar populations. **a-b** Two example sessions showing the progression of optically-induced neural activity in time across electrode contacts following the offset of each laser pulse, in the absence of a visual stimulus. We hypothesize that this delayed activity is a result of local neurons receiving input from the laser-responsive neurons either directly or indirectly. Laser pulse durations for the upper and lower plots were 10 ms and 7 ms, respectively. Differences in mean firing rates between laser and control trials were normalized relative to the total activity during these trials for each channel separately. Responses were binned every 2 ms, and smoothed with a 5 ms Gaussian kernel to improve visualization. In cases where more than one neuron was found on a channel, the response of the most responsive neuron was included. **c** Dimensions of guidetubes. **d** Recording grid schematic. **e-f** Arrangement of optical fiber and recording electrode to minimize distance between devices.

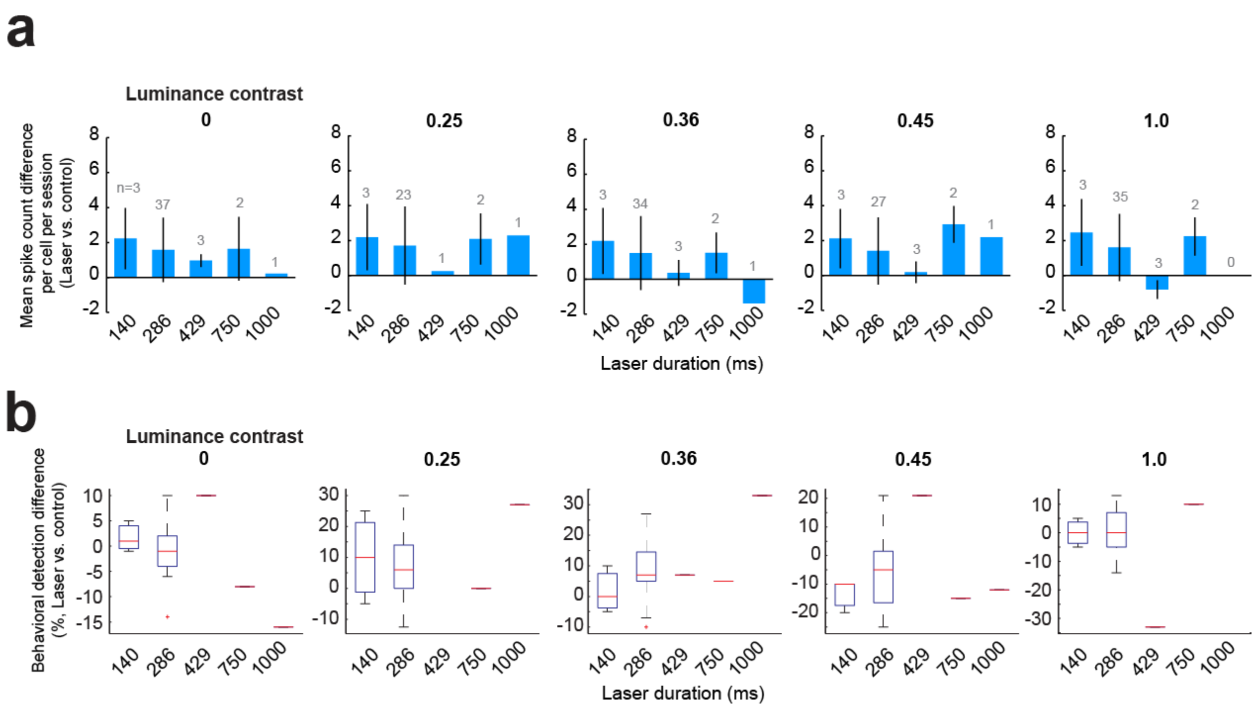

Supplementary Figure 2 Small differences in optical stimulation parameters did not cause systematic differences in firing rates and perceptual detection performance. **a** The mean difference in spike counts for each laser-responsive neuron in the laser vs. control conditions, averaged for each session. The duration of laser stimulation produced no significant difference in the mean spike count change observed across conditions. This is not surprising given that the majority of firing rate change occurs during the first few laser pulses (Fig. 1c-e), and is due to the inactivation kinetics of this variant of ChR2. Error bars show s.e.m. **b** Perceptual contrast detection performance (laser vs. control) for each luminance contrast level (left to right plots) was not systematically altered when the duration of optical stimulation was varied. Boxplots show median (red horizontal line), first and third quartiles. Whiskers show most extreme points and outliers are shown with red asterisks.

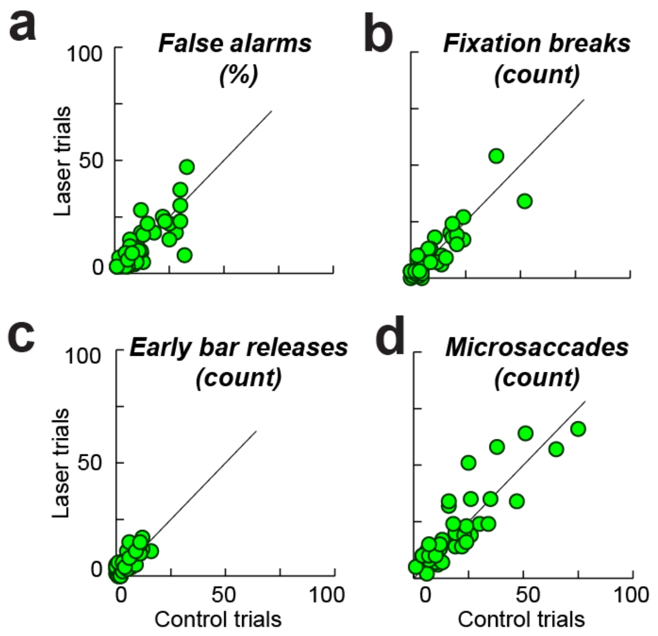

Supplementary Figure 3 Optogenetic stimulation is unlikely to induce phosphenes. **a** False alarm rate – percent incorrect detections reported during no-stimulus trials. Circles represent individual sessions (P=0.45, Wilcoxon signed rank test). **b** Fixation breaks - number of trials aborted in each session due to eye movements outside the fixation window. There was no difference between laser and control trials (P=0.76, Wilcoxon signed rank test). **c** Early bar releases - number of trials aborted in each session due to monkey releasing the response bar before the cued time. There was no difference between laser and control trials (P=0.12, Wilcoxon signed rank test). **d** Microsaccades - total number of microsaccades in each session during a 350-ms window aligned with the start of the laser, or the mean time of the laser for control trials. There was no difference between laser and control trials on any of the above measures (P=0.57, Wilcoxon signed rank test).

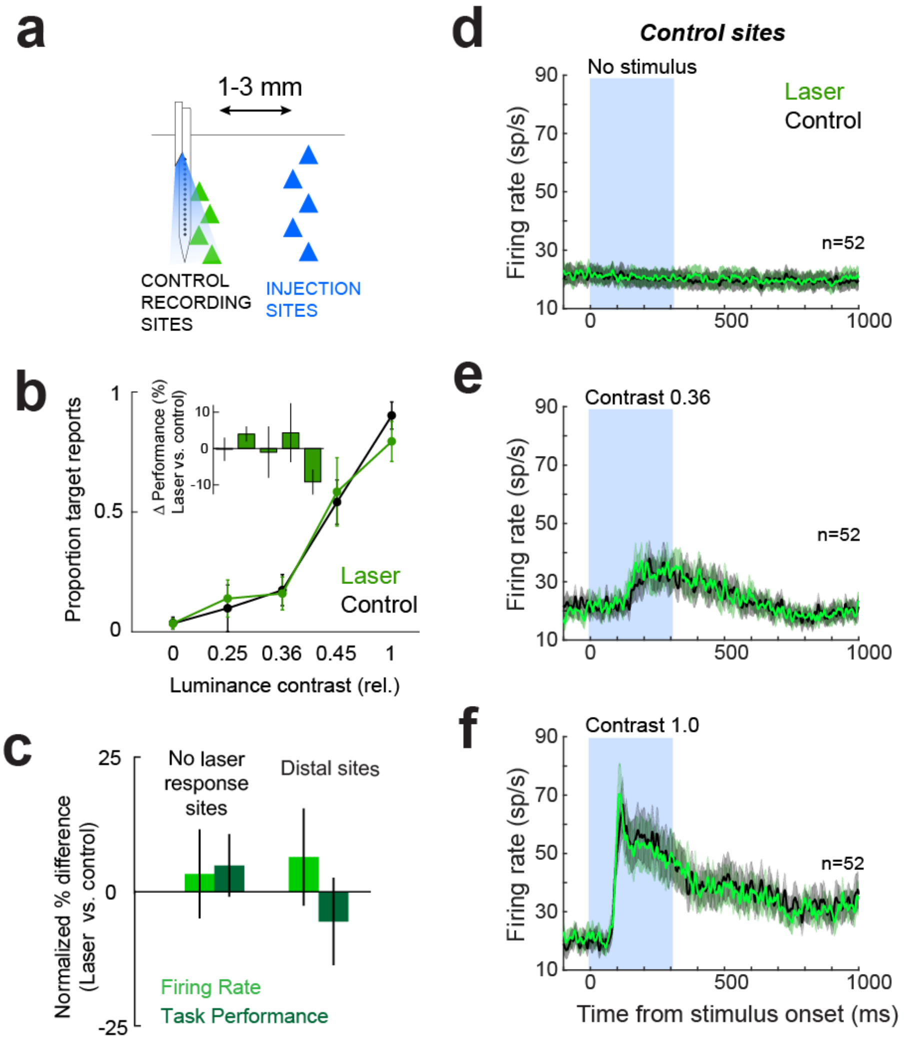

Supplementary Figure 4 Optogenetic stimulation of untransfected cortex. **a** To ensure that the behavioral effects reported here were solely due to optical stimulation (rather than laser-induced local heating, or some other unexpected variable), additional control experiments were performed in which the optical fiber and recording electrodes were positioned distal (1-3 mm) from the nearest injection site. **b** Although cells were tuned to stimulus orientation, there was no significant change in behavioral performance between laser and control trials (P=0.45 Kruskal-Wallis test, df=4, Chi-squared value=3.71). **c** Left group. In addition, even when we recorded near the injection site (4 sessions), when optogenetic stimulation was ineffective (no laser-induced spikes) there was no change in task performance. Right group shows comparable measurements at distal sites. **d-f** As expected, optical stimulation had no effect on neural activity (P>0.05, Wilcoxon ranked sum tests for individual cells across conditions. Errors show s.e.m.

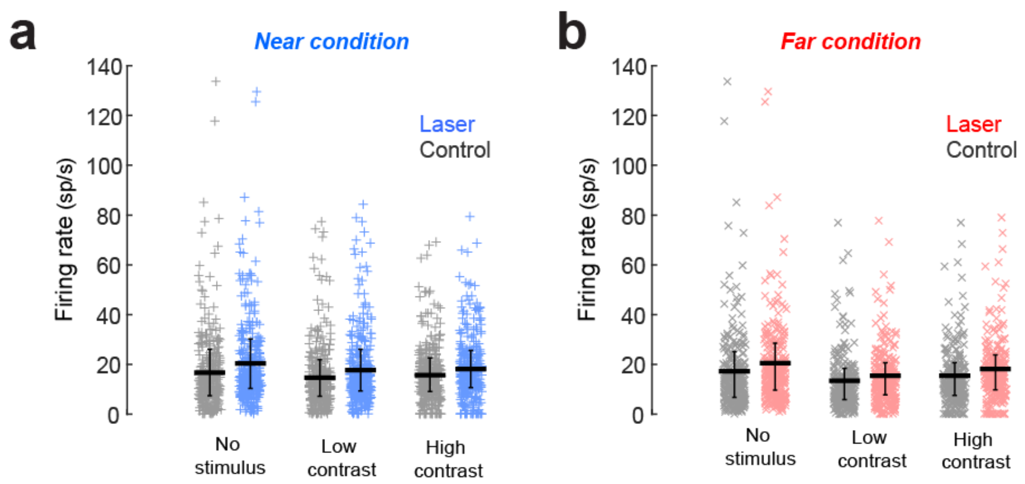

Supplementary Figure 5 Firing rate distributions across populations and stimulus conditions. Firing rate distributions of all laser responsive cells in near conditions (**a**) and far conditions (**b**), for control (gray symbols, left clusters) and laser trials (colored symbols, right clusters). Firing rates were calculated for the first 300 ms aligned with the stimulus onset. Black horizontal line shows the mean. Error bars are ± s.e.m. From left to right, the means ± s.e.m. for each distribution in (a) the 'near' condition are: 16.8 ± 9.3; 20.3 ± 9.9; 14.6 ± 7.4; 17.7 ± 8.4; 15.9 ± 6.7; 18.2 ± 7.4 sp/s (n=329) and (b) the 'far' condition: 16.0 ± 9.2; 19.1 ± 9.5; 12.1 ± 6.2; 14.2 ± 6.5; 14.1 ± 6.6; 16.8 ± 7.1 sp/s (n=268).

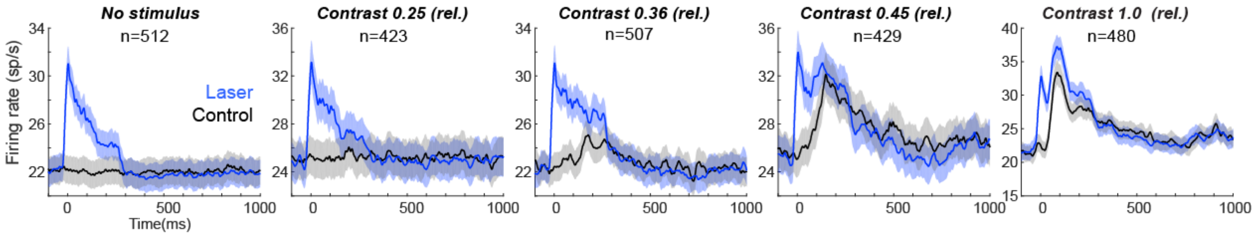

Supplementary Figure 6 Response of the overall population. Not all neurons in each session were modulated by the light. Here we show the responses of the larger cell population that includes all stimulus-driven neurons, regardless of light sensitivity, from all near sessions to stimuli of increasing contrast (increasing from left to right), in both laser (blue) and control (black) conditions. Traces show the mean ± s.e.m. Laser was typically pulsed during the first 300 ms, while the visual stimulus was on for at least 800 ms (see main text for details). The intensity of the light was not modulated across sessions.

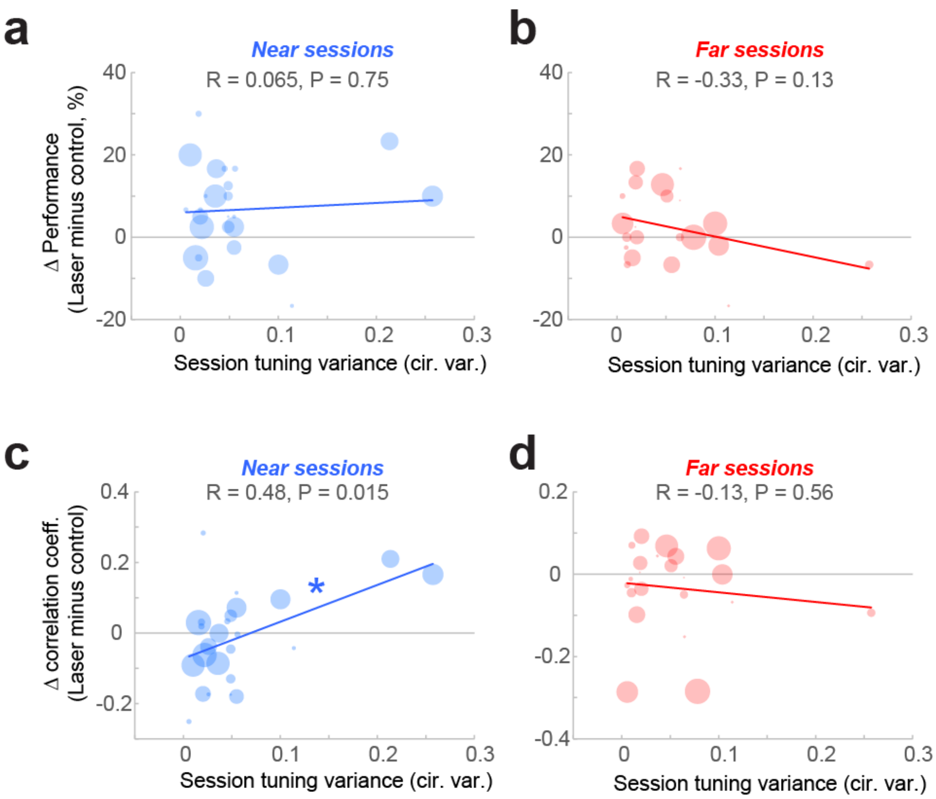

Supplementary Figure 7 Influence of heterogeneity of population orientation tuning preference on perceptual performance and noise correlations. **a-b** Behavior performance changes associated with optogenetic stimulation with respect to the variance of tuning preference of the recorded neural population (calculated as circular variance). Detection performance changes at low contrasts for all 'near' (a), 'far' (b). Marker size indicates number of units in session (range = 2-24 units). Solid lines represent a linear fits. R represent the Pearson correlation coefficient (P is the associated p-value). **c-d** Change in noise correlations associated with optogenetic stimulation depends on similarity of orientation tuning of stimulated cells in 'near' condition (c). Mean difference in noise correlations at low contrasts for all 'near' (c), 'far' (d). Marker size indicates number of pairs in session (range = 1-276 pairs). Solid lines represent a linear fits. R represent the Pearson correlation coefficient (P is the associated p-value). * indicates P<0.05.

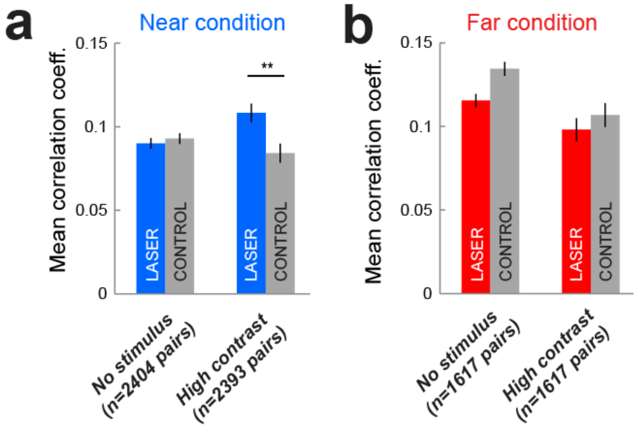

Supplementary Figure 8 Correlation coefficients in other stimulus contrasts for near condition (a) and far condition (b). Error bars show s.e.m. ** P=6.55e-9, Kruskal-Wallis test, posthoc Tukey test.

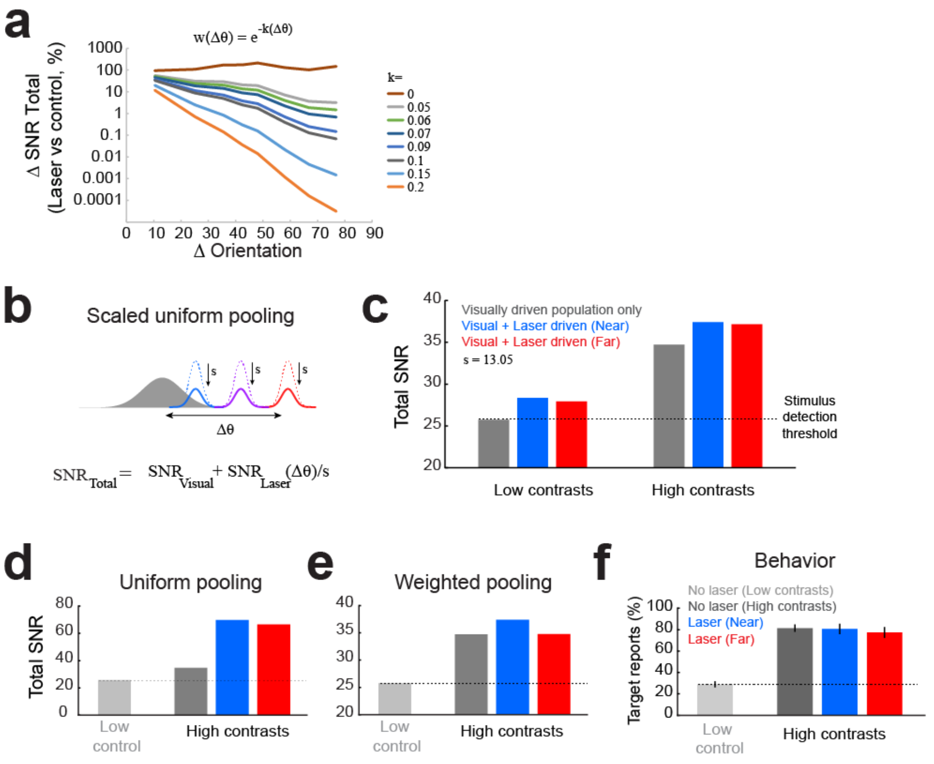

Supplementary Figure 9 Estimating total SNR to predict behavioral performance. **a** In the weighted-pooling model, the contribution of laser-driven neurons is inversely proportional to the orientation-distance to the visually-driven neurons. The figure shows the effect of varying the functional-distance weighting constant, *k*, on total SNR computed for the low contrast condition. Estimates shown in Figs. 5d and 5f used *k*=0.06. **b** Schematic for the scaled uniform pooling model, which aims to take in to account that the number of cells in the light driven population is likely far less than that in the visually driven population. **c** Left group. Scaled uniform pooling results. The visually-driven population is larger (in terms of number of neurons) than the laser-driven population. We asked whether the addition of a scaling factor to the uniform pooling model would result in a total SNR similar to the observed behavioral changes. The scaling constant, *s*, was chosen so that the total SNR resulting from the nearby stimulation (blue) would match that obtained by the functional distance-weighted model in the same condition. The results show that even with the scaled uniform pooling model, the total SNR resulting from stimulation of the 'far' population is above the control condition (and similar to the 'near' condition). **c,d,e** Total SNR for the high contrast condition for the scaled uniform pooling model (c, right), the uniform pooling model (d), and the functional distance-weighted pooling model (e). Visual stimulus and laser conditions include: the visually-driven (no laser) condition (dark gray bar), the 'near' laser condition (blue) or the 'far' laser condition (red). The light gray bars in d and e (far left) show the total SNR in the low contrast condition, for comparison. This was used to estimate the stimulus detection threshold (black horizontal dotted line). For a detection task, it is expected that once the threshold is surpassed, detection will be reliable, and further increasing SNR will not affect detection performance. **f** Behavioral detection performance for low contrast stimuli in the absence of laser stimulation (light gray), and for high contrast stimuli without laser (dark gray), or with 'near' (blue), or 'far' laser stimulation (red). Error bars represent s.e.m. across sessions.
